# Network Integration Analysis and Immune Infiltration Analysis Reveal Potential Biomarkers for Primary Open-Angle Glaucoma

**DOI:** 10.3389/fcell.2021.793638

**Published:** 2021-12-03

**Authors:** Liyuan Wang, Tianyang Yu, Xiaohui Zhang, Xiaojun Cai, He Sun

**Affiliations:** ^1^ Department of Ophthalmology, First Affiliated Hospital of Heilongjiang University of Chinese Medicine, Harbin, China; ^2^ Department of Acupuncture, Second Affiliated Hospital of Heilongjiang University of Chinese Medicine, Harbin, China; ^3^ Department of Ophthalmology, Heilongjiang Provincial Eye Hospital, Harbin, China; ^4^ Department of Endocrinology, Heilongjiang Academy of Sciences of Traditional Chinese Medicine, Harbin, China

**Keywords:** primary open-angle glaucoma, ceRNA, transcription factors, immune infiltration, biomarkers

## Abstract

Primary open-angle glaucoma (POAG) is a progressive optic neuropathy and its damage to vision is irreversible. Therefore, early diagnosis assisted by biomarkers is essential. Although there were multiple researches on the identification of POAG biomarkers, few studies systematically revealed the transcriptome dysregulation mechanism of POAG from the perspective of pre- and post-transcription of genes. Here, we have collected multiple sets of POAG’s aqueous humor (AH) tissue transcription profiles covering long non-coding RNA (lncRNA), mRNA and mircoRNA (miRNA). Through differential expression analysis, we identified thousands of significant differentially expressed genes (DEGs) between the AH tissue of POAG and non-glaucoma. Further, the DEGs were used to construct a competing endogenous RNA (ceRNA) regulatory network and 1,653 qualified lncRNA-miRNA-mRNA regulatory units were identified. Two ceRNA regulatory subnets were identified based on the random walk algorithm and revealed to be involved in the regulation of multiple complex diseases. At the pre-transcriptional regulation level, a transcriptional regulatory network was constructed and three transcription factors (*FOS, ATF4*, and *RELB*) were identified to regulate the expression of multiple genes and participate in the regulation of T cells. Moreover, we revealed the immune desert status of AH tissue for POAG patients based on immune infiltration analysis and identified a specific *AL590666.2*-*hsa−miR−339−5p*-*UROD* axis can be used as a biomarker of POAG. Taken together, the identification of regulatory mechanisms and biomarkers will contribute to the individualized diagnosis and treatment for POAG.

## Introduction

Glaucoma is the main cause of irreversible blindness, which includes several subtypes such as primary, secondary, angle-closure glaucoma and open-angle glaucoma (Weinreb and Khaw, 2004; Youngblood et al., 2019). Among them, primary open-angle glaucoma (POAG) is the most common. The clinical manifestations of POAG include optic nerve damage and loss of retinal ganglion cells, and high blood pressure and increased intraocular pressure are risk factors for POAG. Since the symptoms of POAG appear at a relatively late stage and the blindness caused by it is irreversible, early diagnosis is necessary (Weinreb et al., 2014). The identification of biomarkers is helpful for the early diagnosis of POAG patients (Kokotas et al., 2012). Although there were many studies on the identification of biomarkers of POAG, the limitations of the data have caused the limitations of the experimental results. For example, although Liu et al. found that *hsa-miR-210-3p* can be used as a biomarker of POAG from peripheral blood (Liu et al., 2019), the gene transcript also includes mRNA and long non-coding RNA (lncRNA) and did not reveal the pathogenesis of *hsa-miR-210-3p*.

With the progress of scientific research, the function of non-coding RNA has been unveiled. LncRNA and miRNA, as the two main types of non-coding RNA, have been shown to play an important role in the chromatin reprograming (Anastasiadou et al., 2018) and regulation of gene transcription through the competing endogenous RNAs (ceRNA) regulatory mechanism (Salmena et al., 2011). In the ceRNA network, mRNA and lncRNA act as a miRNA sponge to participate in ceRNA regulation determined by miRNA response elements (MREs) (Zhang et al., 2021). The ceRNA regulatory mechanism plays a role at the post-transcriptional level and is an important method to explain the dysregulation of transcript expression in diseases. For POAG, the construction of ceRNA regulatory network will assist in revealing its pathogenesis.

Over the past decade, the immune microenvironment has been a hot area of biological research, which includes immune infiltration, antigen presentation, immune cell exhaustion and immune cell communication. The immune microenvironment is composed of a variety of lymphocytes, such as T cells, B cells and macrophages, etc. Previous studies have shown that the neuroinflammatory response in POAG patients was thought to be caused by a defective immune response (Vernazza et al., 2020). For example, M1 polarization of macrophages enhances the antigen presentation ability and tissue inflammatory response (Yunna et al., 2020). Therefore, it is necessary to reveal the immune landscape of POAG to reveal its neuroinflammatory response mechanism.

In this study, we collected multiple sets of transcription profiles of aqueous humor (AH) tissues for POAG patients. Through the integrated analysis of the ceRNA competition network and the transcriptional regulatory network, we revealed the mechanism of the transcriptome dysregulation of POAG and the physiological functions that it affects. Immune infiltration analysis revealed the immune landscape of POAG. Additionally, potential biomarkers of POAG were identified based on machine learning algorithms.

## Methods

### Data Acquisition and Pre-Processing

The mRNA and long non-coding RNA (lncRNA) expression profiles of primary open-angle glaucoma (POAG) were downloaded from the Gene Expression Omnibus (GEO) database (accession number: GSE101727 (Xie et al., 2019), platform: GPL21827, Agilent-079487 Arraystar Human LncRNA miarray V4, Table 1). The raw annotation of GPL21827 only supported the sequence data format and not the gene symbol. Therefore, we mapped the sequences of GPL21287 probes to the human genome annotation file release GRCH37 in GENCODE (Harrow et al., 2012) using the R package “Rsubread” (Liao et al., 2019). Next, the average of standardized signal intensities was used to indicate mRNA/lncRNA expression intensity when multiple probes were mapped to the same mRNA/lncRNA. The miRNA expression profile was also obtained from the GEO database (accession number: GSE105269 (Drewry et al., 2018), platform: GPL24158, NanoString nCounter Human v3 miRNA Assay). The normalized miRNA expression matrix was used directly for the analyses (Table 1).

**TABLE 1:** Expression datasets in the study.

GEO accession number	Platform	Type	Tissue	Samples of POAG	Samples of non-glaucoma
GSE101727	GPL21827	LncRNA and mRNA	Aqueous humor (AH)	10	10
GSE105269	GPL24158	miRNA	Aqueous humor (AH)	12	11

### Differential Expression Analysis of mRNA, lncRNA, and miRNA

Differentially expressed RNAs (mRNAs, lncRNAs, and miRNAs) were identified using the Linear Models for Miarray Data (Limma, v3.44.3) package in R (Ritchie et al., 2015). We considered the RNAs with |*log*_*2*_ *FC*| > 1.5 and *p-value* < 0.01 as the differentially expressed RNAs (DEmRNAs, DElncRNAs, and DEmiRNAs).

### Constructing the POAG Associated Competing Endogenous RNA Network

To identify the POAG associated ceRNA relationships, we first recognized the candidate targets of DEmiRNAs based on the experimentally validated miRNA interaction relationships in lncACTdb v2.0 (http://www.bio-bigdata.net/LncACTdb/) (Wang et al., 2019), mirtarbase v2020 (http://miRTarBase.cuhk.edu.cn/) (Chou et al., 2018), and starbase v3.0 (https://starbase.sysu.edu.cn/) (Li et al., 2014). Next, according to the differentially expressed levels, the opposite changing trends between the expression levels of DEmiRNA-DEmRNA/DElncRNA pairs were retained in the AH (down-regulated miRNAs and up-regulated mRNAs/lncRNAs or up-regulated miRNAs and down-regulated mRNAs/lncRNAs). Furthermore, we calculated the *rho* between the expression levels of DElncRNAs and DEmRNAs. The raw *p-values* $\left(P_{r}\right)$ were adjusted by multiple hypotheses using a permutation method. For each mRNA, the expression value was held consistently, and 1,000 random lncRNAs were used to perform the same Spearman’s correlation test, generating a set of 1,000 permutation *p-values* $\left(P_{p}\right)$ . Finally, an empirical *p-value* $\left(P_{e}\right)$ was corrected using the following formula (which introduces a pseudo-count of 1), i.e.

$$P_{e}=\frac{\mathrm{num}\left(P_{p}\leq P_{r}\right)+1}{1001}$$ (1)

The mRNA-lncRNA pairs with the rho>0.6 and $P_{e}<0.01$ were treated as the co-expressed mRNA-lncRNA pairs. Finally, we constructed the ceRNA triplets relationships in POAG by integrating the miRNA-mRNA/lncRNA pairs and the co-expressed mRNA-lncRNA pairs (Wang et al., 2015). ceRNA network was visualized using the Cytoscape (Shannon et al., 2003).

### Network-Based Prioritization of POAG-Related ceRNA Relationships Discovery

To identify the hub nodes in our ceRNA network, we employed the random walk with restart (Köhler et al., 2008). The POAG-related genes contained in the DisGeNet (Piñero et al., 2020) were considered as the seed genes. Performed random walk on the ceRNA network, with a restart probability of 0.7 using the function random walk in the R package RWOAG (Köhler et al., 2008). The nodes with top 30 visitation probabilities were treated as the hub nodes of the network. The ceRNA triplets consisting of hub nodes were considered as the critical ceRNAs relationships.

### Construction of Transcriptional Regulatory Network

First, the immunosuppressive-related genes were collected from DisGeNET (Piñero et al., 2017) (http://www.disgenet.org) and HisgAtlas v1.0 (Liu et al., 2017) (http://biokb.ncpsb.org/HisgAtlas/). In addition, we searched for the keyword “immunosuppressive agents” in the Drugbank (Wishart et al., 2018) database (https://www.drugbank.ca/) and obtained 311 immunosuppressive-related drugs. In total, the 1,332 immunosuppressant-related genes were obtained from the above three databases. Next, the immunosuppressive-related genes in differentially expressed protein coding genes (PCGs) were extracted for the construction of transcriptional regulatory network. Moreover, the regulation data of transcription factors (TF) and target gene for human were downloaded from the TRRUST v2.0 (Han et al., 2018) (https://www.grnpedia.org/trrust/) and ORTI (Vafaee et al., 2016) databases (http://orti.sydney.edu.au/about.html). The TF target gene relationship pairs related to the immunosuppressive-related DEmRNAs were extracted. Further, the Spearman’s correlation coefficient (*rho*) between the genes of each pair was calculated and the cutoff of the *p*-value and *rho* were set to 0.05 and 0.5. Then, we constructed the TF-target network using Cytoscape software. We then analyzed the topological properties of the network and extracted the top 3 genes of degree as key drive factors.

### Functional Enrichment Analysis

To annotated the potential biology functions of differentially expressed genes and ceRNA triplets, we performed functional enrichment analysis on the mRNAs using Metascape (http://metascape.org/gp/index.html) (Zhou et al., 2019). For the mRNA list, pathway and process enrichment analysis have been carried out with the following ontology sources: KEGG Pathway, GO Biological Processes, Reactome Gene Sets, and Canonical Pathways.

### Immune Infiltration Analysis

The pre-processed expression matrix of PCGs for the GSE101727 series was used for immune infiltration analysis using CIBERSORT (Newman et al., 2015). CIBERSORT is a method to characterize the cellular composition of complex tissues from gene expression profiles.

### Statistical Analysis

The ROC curves were performed using the R package pROC. The gene sets enrichment analysis using the Fisher’s exact test. All statistical analysis was performed using the R (v 3.6.2).

## Results

### Differential Expression Analysis Depicts the Transcriptional Features of POAG

**FIGURE 1:**
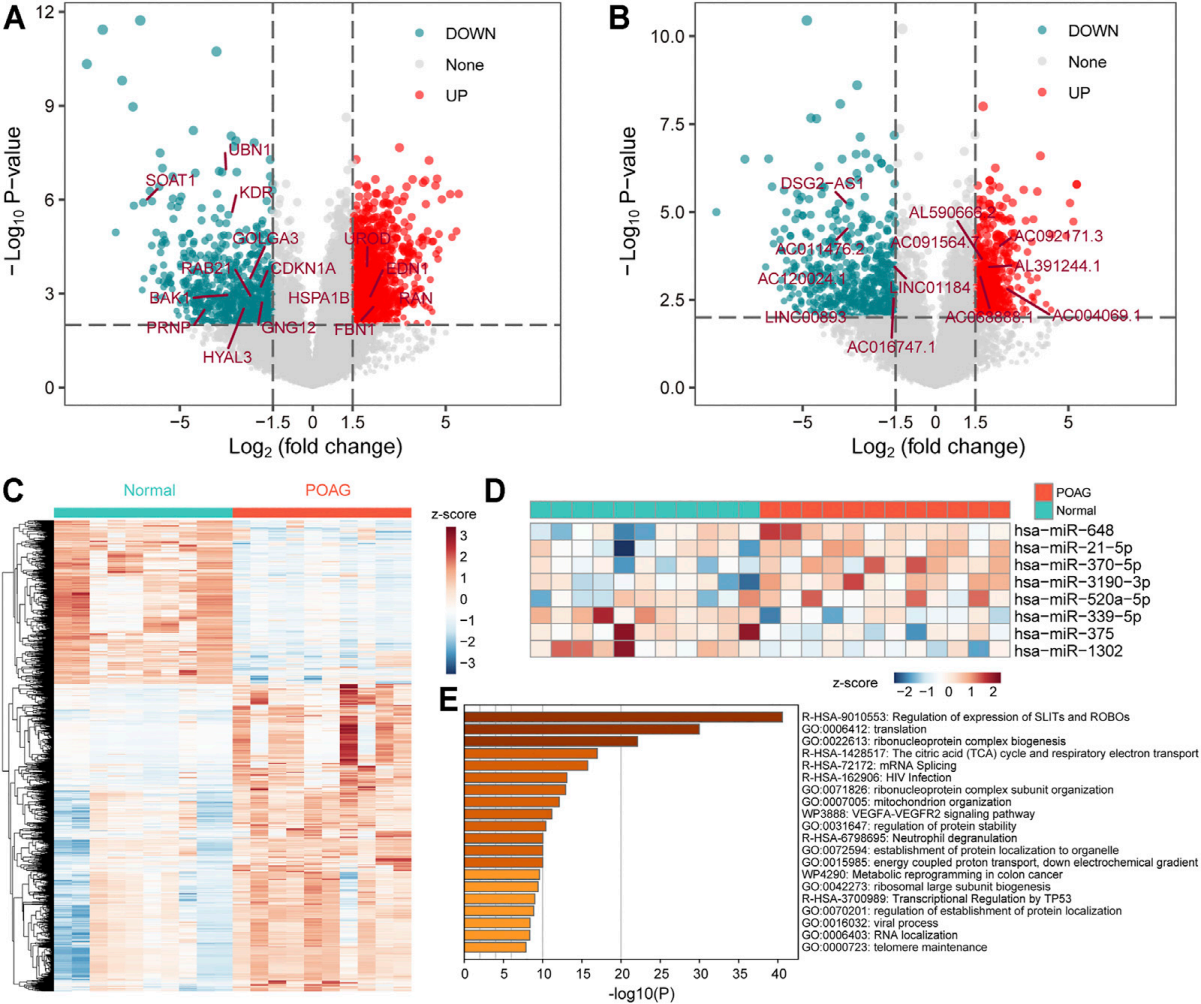
The differentially expressed genes in AH of POAG. **(A,B)** The DEmRNAs and DElncRNAs between the two groups of AH tissue for POAG (GSE101727) and non-glaucoma are displayed by volcano map. The vertical dotted line is 1.5, and the horizontal dotted line is 2. These marked genes are identified in the following hub-subnets of ceRNA network. **(C)** The expression profile of differentially expressed genes (mRNA and lncRNA) is displayed with heat map. The column label shows the sample type. **(D)** The expression profile of the DEmiRNAs between the two groups of AH tissue for POAG (GSE105269) is displayed with heat map. The column label shows the sample type. **(E)** Functional enrichment results of DEmRNAs calculated by Metascape tool are displayed by bar plot. The darker the color, the more significant the enrichment function.

In the regulation of gene expression, transcription is an initial step and one of the most critical steps (Prieto and McStay, 2005). To explore the changes in gene expression of POAG patients at the transcriptional level, the limma algorithm was used to identify genes that were significant differentially expressed in the AH of POAG compared to non-glaucoma. For GSE101727 series, 789 mRNAs were recognized to be down-regulated and 1,487 mRNAs were recognized to be up-regulated in AH (Figure 1A). In addition to considering the expression of protein-coding gene (PCG), the expression of non-coding gene that has been proven to play an important role in the activities of cell (Bridges et al., 2021) was also our focus. Further, there were 576 down-regulated and 614 up-regulated lncRNAs in AH (Figure 1B), which identified in GSE101727 series. Differentially expressed genes were used as features to identify non-glaucoma and POAG samples. We found that non-glaucoma samples and POAG samples can be distinguished and there are significant differences between the two groups (Figure 1C), indicating that the identified DEmRNAs and DElncRNAs can be used as the signature of POAG patients. In the ceRNA competition mechanism, miRNA is a crucial part (Smillie et al., 2018). Therefore, we specifically collected GSE105269 series data to identify DEmiRNA in AH of POAG. We found that 8 miRNAs (3 down-regulated and 5 up-regulated) were significantly differently expressed in AH of POAG (Figure 1D). Moreover, it is necessary to explore which physiological mechanisms these differentially expressed genes affect. The PCGs up-regulated in AH of POAG were used for functional enrichment analysis by Metascape tool. We found that the up-regulated PCGs are significantly enriched in protein synthesis and immune regulation (Figure 1E). Among them, the function of the top enrichment is the regulation of the expression of *SLITs* and *ROBOs*, which has been shown to be involved in the migration and positioning of neuronal precursor cells and the growth of neuronal axons (Tong et al., 2019). All these indicate that the neurons and immune microenvironment of AH in POAG patients have been changed.

### The ceRNA Network Reveal the Mechanism of Gene Expression Variations

**FIGURE 2:**
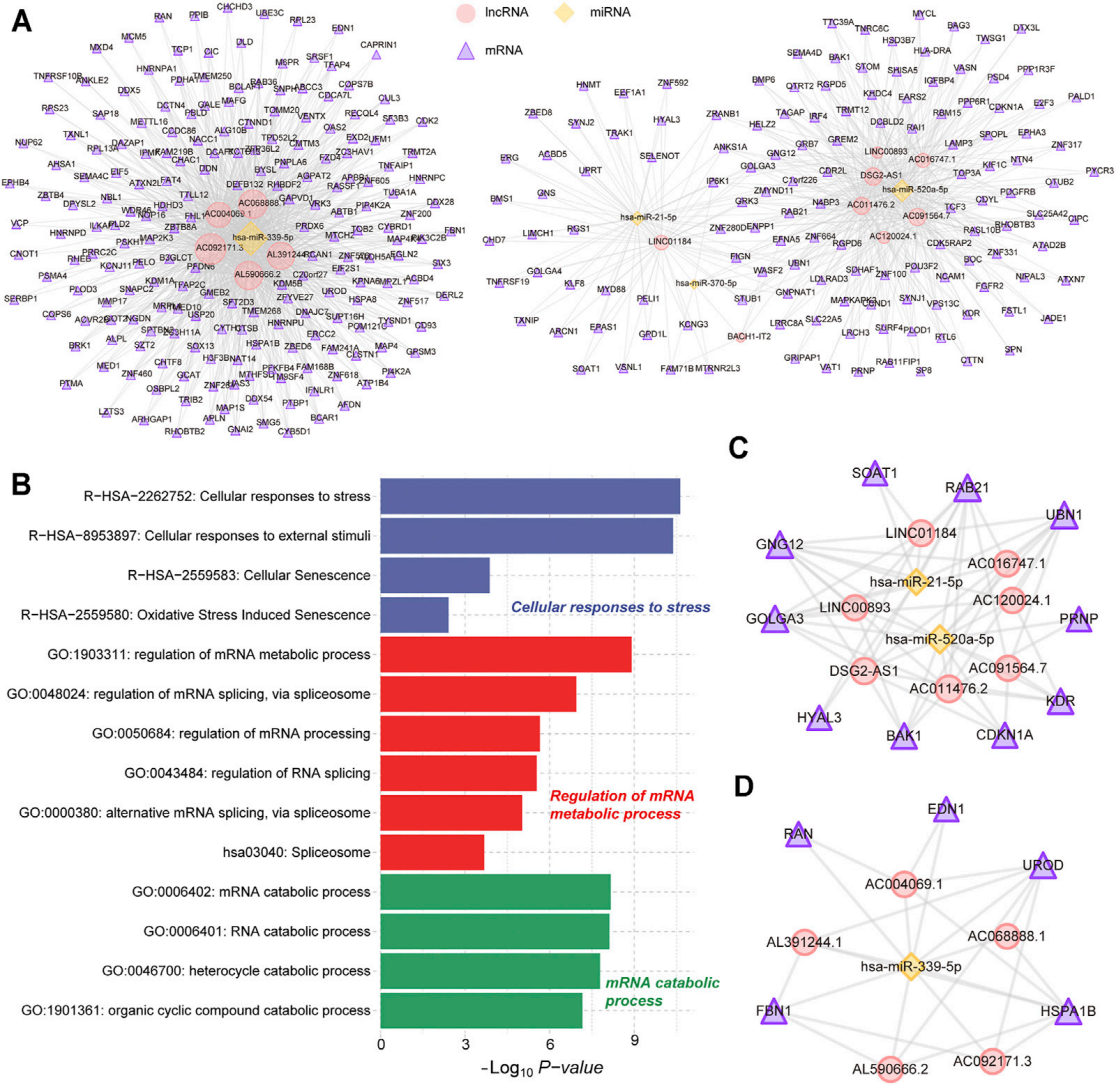
Construction of ceRNA regulatory network. **(A)** The POAG-related ceRNA triplets’ network in AH tissue. The yellow nodes represent the miRNAs. The red nodes represent the lncRNAs. The purple nodes represent the mRNAs. **(B)** Fuctional enrichment results of mRNAs in ceRNA networks are displayed by the bar plot. Three different colors represent three function sets. **(C,D)** The hub POAG-related ceRNA triplets’ sub-network in AH tissue. The yellow nodes represent the miRNAs. The red nodes represent the lncRNAs. The purple nodes represent the mRNAs.

The ceRNA regulatory mechanism plays an important role in the post-transcriptional regulation of genes. Using the ceRNA network to reveal the regulatory mechanisms of differentially expressed genes in AH tissue was conducive to the pathogenesis of POAG. Through the screening of genes involved in ceRNA regulation (see methods), we have identified 4 miRNAs that can bind to 13 lncRNAs and regulate their expression. Furthermore, the 4 miRNAs can regulate the expression of 333 mRNAs and then constitute 1,653 lncRNA-miRNA-mRNA regulatory units (Figure 2A). Through functional enrichment analysis, we found that the genes involved in ceRNA regulation are significantly enriched in cellular responses to stress, regulation of mRNA metabolic process and mRNA catabolic process (Figure 2B), indicating that the ceRNA mechanism in AH tissue of POAG could affect specific physiological mechanisms by regulating gene expression. Further, the hub nodes in the ceRNA network were identified by restarting the random walk algorithm (see methods). The ceRNA triad composed of hub nodes constitutes two ceRNA subnets. The ceRNA subnet_1 consists of 7 lncRNAs, 2 miRNAs and 10 mRNAs (Figure 2C). Among them, *hsa-miR-21-5p* as a ceRNA has been proven to play an important role in multiple complex diseases (Xiong et al., 2020; Jiang et al., 2020). The ceRNA subnet_2 consists of 5 lncRNAs, 1 miRNA and 5 mRNAs (Figure 2D). We found that *FBN1* regulated by *hsa-miR-339-5p* is the causative gene of a variety of genetic diseases, including fibrinopathy and Marfan syndrome (Sakai et al., 2016). The genetic polymorphism of *HSPA1B* was closely related to the disorder of neuroregulation (Bosnjak Kuharic et al., 2020). Taken together, these suggest that the ceRNA regulatory mechanism plays an important role in AH tissue of POAG.

### Key Factors Driving the Progress of POAG

**FIGURE 3:**
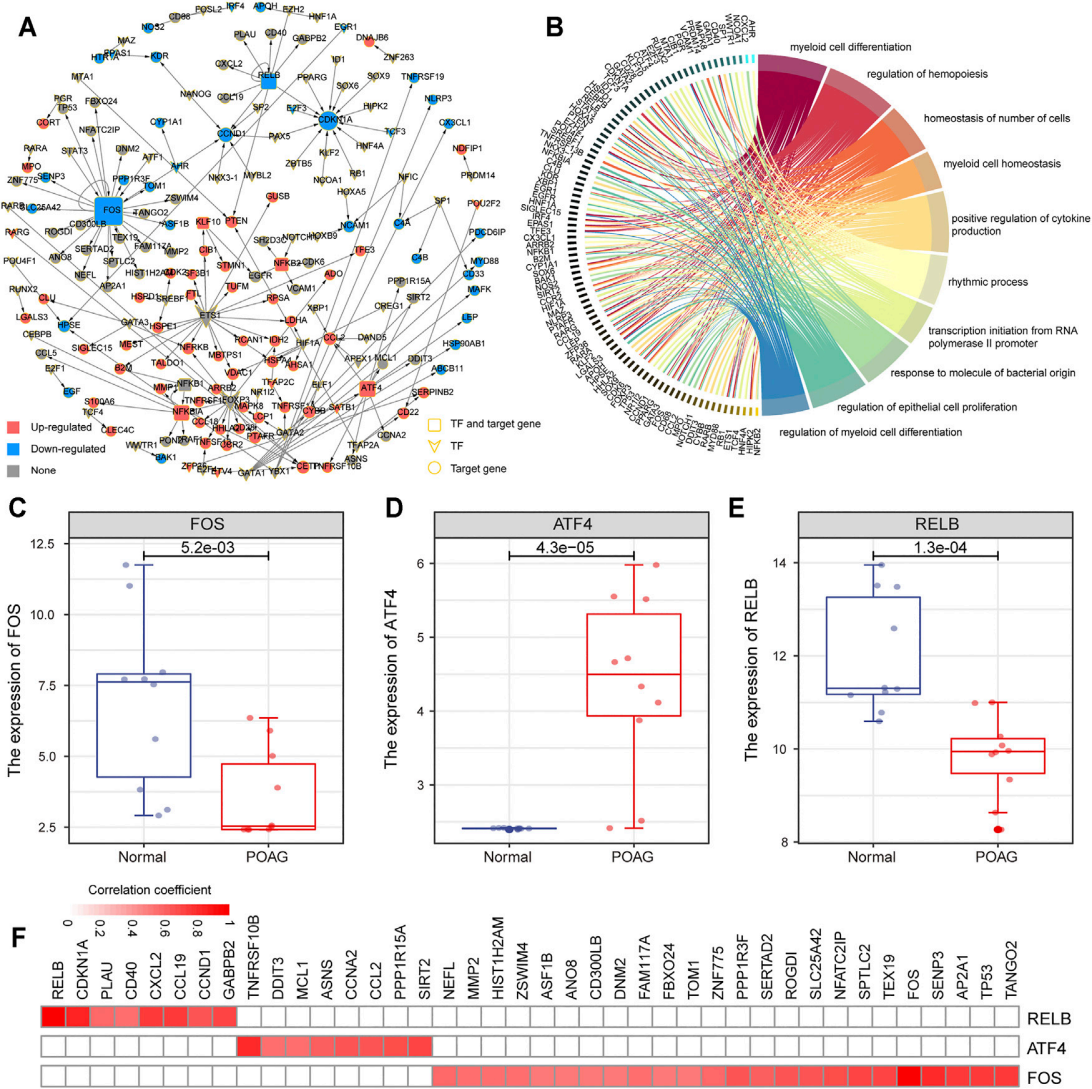
Construction of transcriptional regulatory network. **(A)** The POAG-related transcriptional regulatory network in AH. The square represents the TF and target gene, the diamond represents TF, and the circle represents the target gene. Red color means up-regulation of gene expression, blue color means down-regulation. **(B)** Function enrichment results of mRNAs in transcriptional regulatory networks are displayed by the circle plot. The right side of the panel represents the function, and the left side represents the genes enriched in the function. **(C–E)** The expression of 3 TF (*FOS, ATF4,* and *RELB*) between AH tissue of POAG and non-glaucoma is shown by boxplot. **(F)** The correlation between the 3 TF (*FOS, ATF4,* and *RELB*) and its target genes. The color changes with the variation of the correlation coefficient.

The TFs regulate the initiation and intensity of transcription of specific genes, which is an important driving factor in life activities (Lambert et al., 2018). To identify the driving factors that play an important role in POAG, a transcriptional regulatory network was constructed based on differentially expressed PCGs in GSE101727 series. By combining the previous research data and the correlation analysis of gene expression, 181 TF-target gene units were identified and the transcriptional regulatory network was constructed (Figure 3A). The network contained 73 TFs and 116 target genes. Further, functional enrichment analysis was used to explore the physiological mechanism involved in this transcriptional regulatory network. We found that the genes in this transcriptional regulatory network are significantly enriched in the regulation of myeloid cell differentiation and cell proliferation (Figure 3B), indicating that the specific expression of TF drives the expression of target genes and affects the immune microenvironment of POAG. Moreover, we identified the top 3 TFs (*FOS, ATF4*, and *RELB*) of degree as a key driver in the transcriptional regulatory network (Figures 3C‐E). The *FOS* and *RELB* were significantly down-regulated and *ATF4* was significantly up-regulated (Figure 3D) in AH of POAG. Studies have shown that *RELB* plays a key role in the development of T cells and controls the proliferation of T cells, indicating that changes in *RELB* expression may be related to variations of the immune microenvironment for POAG (Zhou et al., 2020). Besides, we found that *CDKN1A* has the highest correlation with *RELB* at the transcript level (Figure 3F), and *CDKN1A* encodes cyclin which is regulated by kinase inhibitors (El-Deiry, 2016), indicating that *RELB* may control the proliferation of T cells by regulating the expression of *CDKN1A*. All these indicate that there are several driving factors that play an important role in the changes in the physiological mechanism of POAG.

### Immune Infiltration Characteristics of POAG

**FIGURE 4:**
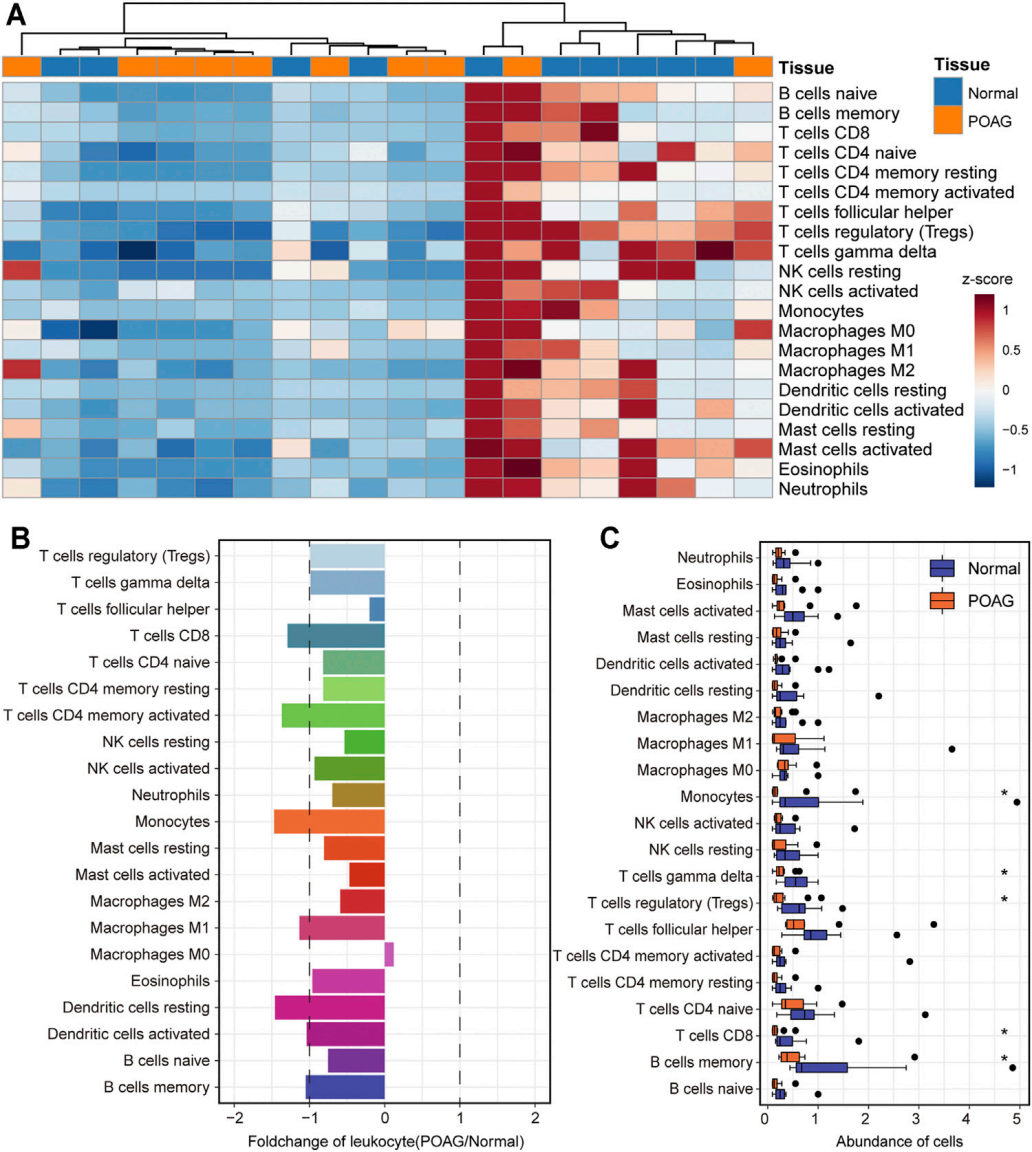
The landscape of immune cell infiltration for POAG. **(A)** The fraction of immune cells in the AH tissue of POAG and non-glaucoma is shown by heatmap. The column label shows the sample type. **(B)** The fold change of the fraction of immune cells between the AH tissue of POAG and non-glaucoma. The two vertical dashed lines represent 1 and -1 respectively. **(C)** The fraction of immune cells in the AH tissue of POAG and non-glaucoma is shown by boxplot. Wilcoxon rank sum test is used to calculate the statistical difference between the two groups.

The dynamics of the immune microenvironment is an important feature of the occurrence and development of diseases (Makowski et al., 2020). Identifying the immune characteristics is conducive to enriching the exploration of the pathogenesis of POAG. Therefore, the CIBERSORT tool was used to calculate the immune cell composition of each AH samples of POAG and non-glaucoma samples through the deconvolution algorithm. After preprocessing the immune cell fraction matrices, the consensus clustering algorithm was used to identify the distance between samples including AH of POAG and non-glaucoma samples. We found that POAG and non-glaucoma individuals can be distinguished by immune cell components and POAG individuals are in an immune desert state (Figure 4A). Further, the statistical test was used in the analysis of the difference between the immune cell components of the AH of POAG and non-glaucoma sample. From the perspective of the fold changes of immune cell components, the CD8^+^ T cell, CD4^+^ memory T cell, monocytes, macrophages, M1 and dendritic cell components of POAG individuals are significantly different from those of non-glaucoma individuals (Figure 4B). Besides, the Wilcoxon rank sum test was used to test the significance of differences in immune cell components between the two groups. We found that monocytes, γδ T cells, Tregs, CD8^+^ T cells and memory B cell components are significantly different between POAG and non-glaucoma individuals (Figure 4C). Monocytes, as a kind of myeloid cells, play an important role in presenting antigens in the organism and their fraction was significantly down-regulated in POAG, which may be an important sign of POAG patients. POAG is a neurodegenerative disease and neuroinflammation occurs during its pathogenesis (Weinreb and Khaw, 2004; Evangelho et al., 2019), which may be related to the lack of Treg. Taken together, these suggest that the AH of POAG is in an immune desert state and the significant down-regulation of specific immune cell components can be used as the marker of POAG.

### Biomarkers of POAG

**FIGURE 5:**
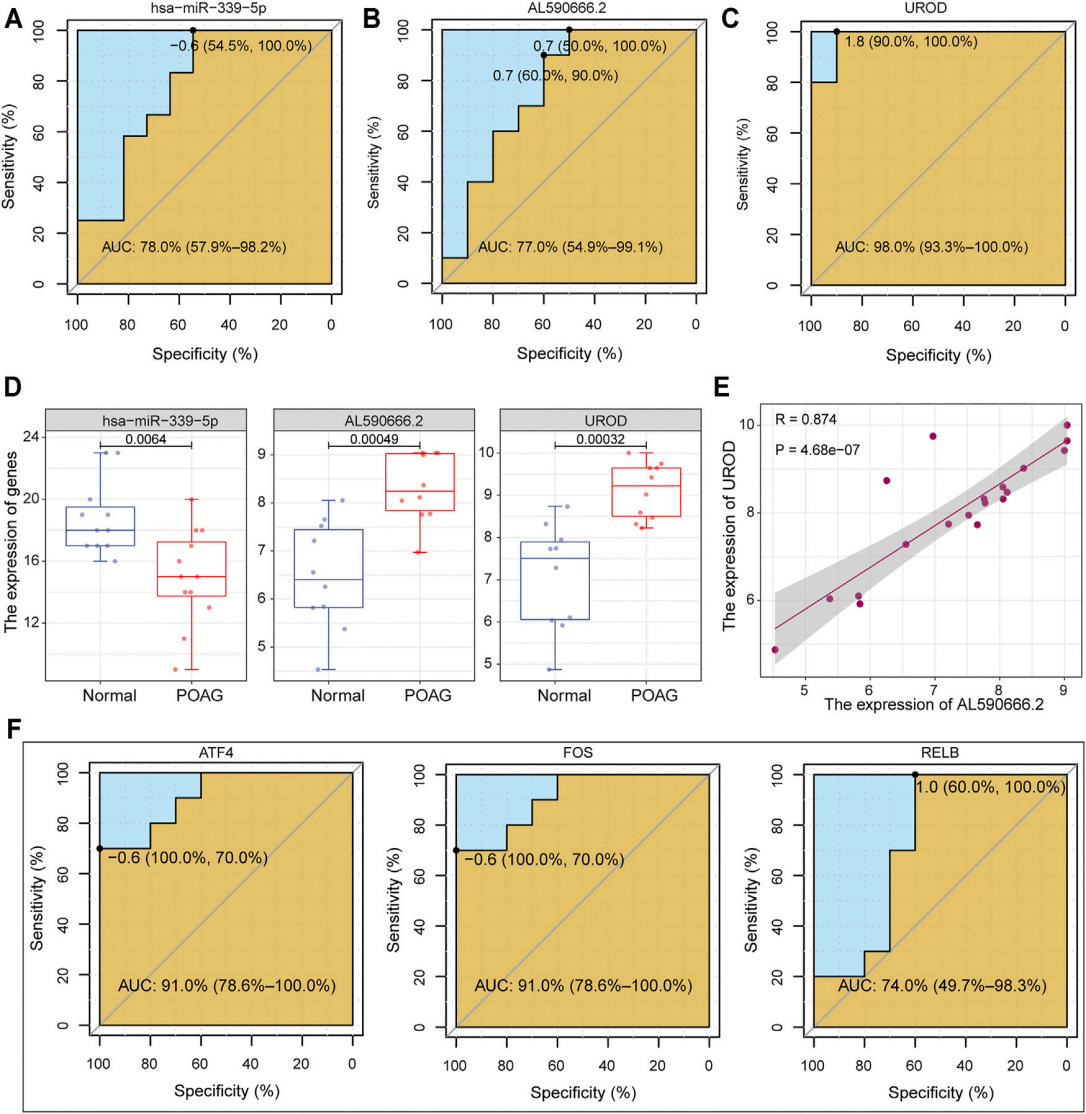
Machine learning was used to identify POAG biomarkers. **(A–C)** The potential of *has-miR-339-5p, AL590666.2,* and *UROD* in identifying POAG diseases is depicted by the ROC curve. The AUC values are calculated. **(D)** The expression of *has-miR-339-5p, AL590666.2,* and *UROD* in AH tissues of POAG and non-glaucoma. Wilcoxon rank sum test is used to calculate statistical differences. **(E)** The correlation between AL590666.2 and UROD in expression. **(F)** Same as in **(A–C)** but for the ATF4, FOS, and UROD.

The identification of biomarkers of POAG is helpful for its clinical diagnosis and treatment. Therefore, we collected important genes in the ceRNA regulatory subnet and drive factors identified above. For the two ceRNA regulatory subnet, the genes in each lncRNA-miRNA-mRNA unit were used as features to distinguish POAG from non-glaucoma individuals and the ROC curve was used to evaluate the stability of the feature. The *AL590666.2*-*hsa−miR−339−5p*-*UROD* axis was recognized to be able to stably distinguish between POAG and non-glaucoma individuals. Among them, *UROD* has the highest AUC value of 0.98 compared to 0.77 of *AL590666.2* and 0.78 of *hsa−miR−339−5p* (Figures 5A–C). Uroporphyrinogen decarboxylase encoded by *UROD* was an important element in hemoglobin synthesis, which is significantly up-regulated in POAG (Figure 5D). Studies have shown that patients with POAG have red blood cell backlog and high plasma specific viscosity (Wang et al., 2004; Xu et al., 2020), indicating that the up-regulation of *UROD* may be an important cause of blood deformation in POAG patients. Further, we found that AL590666.2 and UROD have a strong correlation (Figure 5E), suggesting that AL590666.2 may be an important biomarker of POAG. For the top three TFs identified above, the AUC values of ATF4, FOS, and RELB were 0.91, 0.91, and 0.74, respectively (Figure 5F). All these suggest that these genes can be used as biomarkers of POAG for clinical diagnosis and treatment.

## Discussion

In this work, we have integrated multiple sets of transcript data (mRNA, lncRNA, miRNA) and revealed important functional subnets and driving factors of POAG through ceRNA competition network and transcriptional regulatory network analysis. Through statistical testing, thousands of genes (DEmRNA, DElncRNA, DEmiRNA) differentially expressed in POAG’s AH tissue have been identified. We have identified 1,653 lncRNA-miRNA-mRNA regulatory units and two functional subnets in AH tissue, which will help reveal the pathogenesis of POAG. Further, the transcriptional regulatory network was constructed based on differentially expressed genes and 3 TFs were recognized to play an important role in the transcriptome disorder of the POAG’s AH tissue. We have used the CIBERSORT tool and transcription profile of AH tissue to reveal the immune landscape of POAG. We found that the components of immune cells in AH tissue of POAG were globally down-regulated compared to non-glaucoma. Additionally, a ceRNA regulatory axis (*AL590666.2*-*hsa−miR−339−5p*-*UROD*) and 3 TFs (*ATF4, FOS*, and *RELB*) have been identified as potential biomarkers for POAG patients.

POAG is the most common form of glaucoma disease, which is a disease of the optic nervous system and causes irreversible blindness (Li et al., 2016). The identification of POAG’s biomarkers is the direction of the efforts of many researchers. For example, Liu et al. identified *hsa-miR-210-3p* in peripheral blood as a biomarker of POAG based on miRNA expression profile (Liu et al., 2019). However, polygene dysregulation and interaction were the inherent causes of POAG. We have revealed the regulatory relationship of dysregulated genes and the pathogenesis of POAG through multi-network integration analysis. The *hsa-miR-21-5p* and *FBN1* were the key genes in the ceRNA regulatory subnet that we have identified, which play an important role in multiple complex diseases.

For our identified driving factor ATF4, it has been confirmed in previous studies that it can cause glaucoma by promoting ER client protein load (Kasetti et al., 2020) and regulating trabecular meshwork remodeling (Zhao et al., 2020). In addition to common transcriptome analysis, Liu et al. identified F-box protein (FBOX) and vaccinia-associated kinase 2 (VRK2) that may interact with tumor protein p53 (TP53) to regulate apoptosis and play a negative role in POAG from the perspective of genetic lineage (Liu et al., 2012). Among them, *FBOX* and *TP53* were target genes of the key TF *ATF4* and *FOS* identified in this work. Moreover, several potential biomarkers of POAG were revealed through integrated network analysis in this work.

Among identified protein-coding genes that are significantly differentially expressed in the AH tissue of POAG, *RELB* and *CDKN1A* were a pair of important transcriptional regulatory units. The RELB has been confirmed in previous studies to regulate the proliferation of T cells (Zhou et al., 2020) and the expression of its target gene CDKN1A was closely related to the cell cycle (El-Deiry, 2016). Therefore, the down-regulation of CDKN1A expression mediated by RENL may be an important reason for the decreased level of T cell infiltration. Red blood cell backlog and high plasma specific viscosity were an important physiological manifestation of POAG. This trait may be related to the up-regulation of *UROD* expression.

## Conclusion

In conclusion, we integrated multi-network analysis to identify important functional subnets and driving factors, which will help advance the research on the pathogenesis of PAOG. Immune infiltration analysis and feature recognition reveal the immune desert state and the biomarkers of POAG. Taken together, our research provides theoretical guidance for the clinical diagnosis and treatment for POAG.

## Data Availability

Publicly available datasets were analyzed in this study. This data can be found here: GSE101727 and GSE105269 from the Gene Expression Omnibus (GEO) database.
